# A co-fermentation strategy to consume sugar mixtures effectively

**DOI:** 10.1186/1754-1611-2-3

**Published:** 2008-02-27

**Authors:** Mark A Eiteman, Sarah A Lee, Elliot Altman

**Affiliations:** 1 Center for Molecular BioEngineering, Department of Biological and Agricultural Engineering, University of Georgia, Athens, GA 30602, USA

## Abstract

We report a new approach for the simultaneous conversion of xylose and glucose sugar mixtures into products by fermentation. The process simultaneously uses two substrate-selective strains of *Escherichia coli*, one which is unable to consume glucose and one which is unable to consume xylose. The xylose-selective (glucose deficient) strain *E. coli *ZSC113 has mutations in the *glk*, *ptsG *and *manZ *genes while the glucose-selective (xylose deficient) strain *E. coli *ALS1008 has a mutation in the *xylA *gene. By combining these two strains in a single process, xylose and glucose are consumed more quickly than by a single-organism approach. Moreover, we demonstrate that the process is able to adapt to changing concentrations of these two sugars, and therefore holds promise for the conversion of variable sugar feed streams, such as lignocellulosic hydrolysates.

## Background

The efficient and simultaneous conversion of pentoses and hexoses is a significant hurdle to the economic utilization of biomass hydrolysates for the generation of any fermentation product. A recent review noted "the lack of a microorganism able to ferment efficiently all sugars released by hydrolysis from lignocellulosic materials has been one of the main factors preventing utilization of lignocellulose" [1]. The central problem is that either the desired microorganism consumes this sugar mixture sequentially (e.g. first glucose and then xylose) or the organism is unable to utilize the pentose at all (e.g. *Saccharomyces cerevisiae*). Although the inability of microorganisms to utilize xylose effectively is most commonly associated with fuel ethanol production, the formation of other fermentation products (butanol, succinic acid, lactic acid, pyruvic acid, etc.) from sugar mixtures could also benefit from a strategy to use both sugars effectively.

Essentially two approaches have been applied to ameliorate the problem of simultaneous pentose and hexose consumption. One strategy has been to introduce genes involved in xylose consumption into an organism which does not natively have this ability but can generate a desirable product. For example, although it does not naturally consume xylose, the common yeast *Saccharomyces cerevisiae *is the most widely used organism for ethanol production. Researchers have long sought to incorporate xylose-consuming genes into this organism, and the xylose reductase, xylitol dehydrogenase and xylulokinase genes fused to glycolytic promoters have been successfully integrated into the yeast chromosome [2,3]. A second strategy is to alter the cellular machinery preventing xylose consumption in the presence of glucose. For example, a *ptsG *mutation in an *E. coli *ethanol production strain reduces the glucose-mediated repression of xylose consumption [4]. The underlying goal for both strategies for consuming sugar mixtures has been to develop a single organism that can do it all.

Strategies which require a single organism to convert xylose and glucose simultaneously suffer from several limitations. One limitation is that despite the presence of the genetic apparatus to consume both sugars, glucose remains the preferred substrate, and the consumption of the sugars is asynchronous. In batch culture with the *E. coli *strain K011 grown on hemicellulose hydrolysate, for example, only 11% of the xylose was consumed after 24 h, while 80% of the glucose was consumed [5]. Though removal of the *ptsG *improves xylose consumption in the presence of glucose, 40% of the xylose remains when the glucose is depleted [4]. Similarly, genetically engineered *S. cerevisiae *containing genes to consume xylose still consumed less than 25% of the xylose when glucose was depleted [3]. Even when xylose isomerase activity was added to *S. cerevisiae *to convert xylose to xylulose extracellularly, 75% of the xylose still remained after the glucose was completely consumed [6].

Microorganisms that consume sugars such as glucose and xylose sequentially must have lower productivities for the generation of a product than if the organism were to consume the sugars simultaneously [1]. Even if an organism could consume the two sugars simultaneously, the ratio of the rates of sugar consumption might fall within a fairly narrow range. Microorganisms that possess a narrow ratio of glucose and xylose consumption could have particular difficulty with a source having a variable sugar concentration, such as biomass hydrolysates, since if the ratio of sugar concentrations falls outside of the organism's range, one sugar will inevitably be partially unconsumed. Essentially, a single microorganism may not be able to adjust the rate of consumption to two substrates in order to match fluctuating sugar concentrations. Therefore, one desirable characteristic of a process to handle sugar mixtures which vary in composition is to self-adjust to that changing concentration. Another desirable characteristic is a process which is stable over the course of time. A single-organism approach can have difficulty in achieving this objective: for example, a chemostat study demonstrated that the presence of both sugars caused a gradual increase in the by-product acetate, which ultimately led to a 20% decrease in ethanol yield [7]. Finally, the metabolic pathways to convert a hexose into a desired product at optimal yield and productivity might not correspond to the metabolic pathways to convert a pentose into the same product. Preferably, a process converting xylose and glucose simultaneously into any product would make these pathways independent of one another, with glucose metabolism not influencing xylose metabolism and vice versa.

We propose a different strategy for the efficient co-utilization of sugar mixtures. The concept centers on the fact that we can readily "design" a single strain that will only utilize, for example, xylose or glucose. Such a strain has "substrate-selective uptake" since it is selective in what compound it is able to metabolize. By deleting a key gene in the xylose metabolic pathway, a strain of *E. coli *can be constructed which is unable to consume xylose. The gene must be selected so that xylose metabolism is eliminated, but also the accumulation of a toxic intermediate must be avoided. Placed in a culture containing xylose and glucose, such a strain should be completely unaffected by the presence of xylose. Similarly, a strain can be constructed which is unable to consume glucose. Placed in a culture with xylose and glucose, such a strain would not utilize the glucose and only consume xylose. The metabolic pathways of each strain could be further designed to optimize yield and productivity of a particular product. Placed simultaneously in a bioreactor containing glucose and xylose, each strain would be expected to act optimally on just one of these sugars and be unaffected by the presence of the other sugar or the other organism.

The goal of this study was to characterize xylose-selective and glucose-selective strains of *Escherichia coli*. Specifically, we set out to construct *E. coli *strains which independently will only consume xylose or glucose without any loss in consumption rate and which can be used together, acting in concert to consume sugar mixtures effectively.

## Methods

### Strains

*Escherichia coli *strains MG1655 (wild-type, F-, λ-), CGSC5457 (ZSC113, *lacZ*827(UGA) or *lacZ*82(Am) *ptsG*22 *manZ*12 *glk*-7 *relA*1 *rpsL*223(strR) *rha*-4), DY330 (Δ*lac*U169 *gal*490 λc1857 Δ(*cro-bioA*)), and ALS1008 (MG1655 *xylA*::Tet) were used in this study. CGSC5457 (ZSC113) was obtained from the *E. coli *Genetic Stock Culture (Yale University). DY330 contains a lambda cI857 lysogen and is used to induce the genes which constitute the lambda Red recombination system [8].

### Generating the xylA::Tet knockout

The *xylA *gene which encodes D-xylose isomerase was knocked out using the lambda Red recombination system. Primers were designed which could amplify the *tetA *gene and promoter from pWM41 (9) bracketed by the first and last 50 bases of the *xylA *coding sequence. The *tetA *gene codes for the tetracycline resistance protein. The forward primer 5' ATGCAAGCCTATTTTGACCAGCTCGATCGCGTTCGTTATGAAGGCTCAAAACATCTCAATGGCTAAGGCG 3' contains the first 50 bases of the *xylA *coding sequence followed by bases 1349 – 1368 of TRN10TETR (Accession Number J01830) from pWM41 while the reverse primer 5' TTATTTGTCGAACAGATAATGGTTTACCAGATTTTCCAGTTGTTCCTGGCGGCTGGTTTATGCATATCGC 3' contains the last 50 bases of the *xylA *coding sequence followed by bases 3020 – 3039 of TRN10TETR from pWM41. The bases from pWM41 are underlined in the primers. The two primers were used to amplify a 1,791 bp fragment from pWM41 DNA using the polymerase chain reaction (PCR) with *Pfu *polymerase. The resulting DNA was gel-isolated and electroporated into DY330 electrocompetent cells which were prepared as described [8]. Tet(R) colonies were then selected. The presence of the *xylA*::Tet knockout was confirmed by the inability of DY330 *xylA*::Tet to grow in minimal xylose media. ALS1008 (MG1655 *xylA*::Tet) was constructed by transducing *xylA*::Tet from DY330 into MG1655 using P1 transduction.

### Growth conditions

For each bioreactor experiment, a single strain was first grown in a tube containing 10 mL BXG medium, then 5 mL transferred to 50 mL BXG medium in a 250 mL shake flask. All flasks were incubated at 37°C and 250 rpm (19 mm pitch). For those fermentations in which a single strain was used, when the OD of the shake flask culture reached approximately 4, the contents of the shake flask were diluted with BXG medium so that 100 mL having an effective OD of 2.0 was used to inoculate the fermenter. For those experiments in which two strains were used in a single fermentation, the contents of two shake flasks were diluted with BXG medium to 100 mL so that each strain had an effective OD of 2.0 (i.e., in the 100 mL volume). Basal medium contained (per L): 13.3 g KH_2_PO_4_, 4.0 g (NH_4_)_2_HPO_4_, 1.2 g MgSO_4_·7H_2_O, 13.0 mg Zn(CH_3_COO)_2_·2H_2_O, 1.5 mg CuCl_2_·2H_2_O, 15.0 mg MnCl_2_·4H_2_O, 2.5 mg CoCl_2_·6H_2_O, 3.0 mg H_3_BO_3_, 2.5 mg Na_2_MoO_4_·2H_2_O, 100 mg Fe(III)citrate, 8.4 mg Na_2_EDTA·2H_2_O, 1.7 g citric acid, and 0.0045 g thiamine·HCl. BXG medium comprised basal medium with 15 g/L glucose and 8 g/L xylose. Shake flask media were adjusted to a pH of 7.0 with 20% NaOH.

### Fermentation

Batch experiments were carried out in a 2.5 L bioreactor (Bioflow 2000, New Brunswick Scientific Co. Edison, NJ, USA) containing 1.0 L BXG medium. Throughout aerobic growth, air was sparged into the fermenter at a flowrate of 1.0 L/min, and the agitation was 1000 rpm to ensure no oxygen limitation. In some experiments an anaerobic phase was initiated after aerobic growth. For these cases additional xylose and/or glucose was supplied as reported. To maintain anaerobic conditions, carbon dioxide was provided at a flowrate of 0.2 L/min, and the agitation was 150 rpm.

Fed-batch experiments were carried out in the same vessel initially containing 1.0 L basal medium (no xylose and glucose). Immediately after inoculation, a feed containing a mixture of xylose and glucose without additional medium components commenced as reported. This medium was fed at an exponentially increasing rate designed to achieve a growth rate of 0.1 h^-1 ^for a substrate concentration of 30 g/L.

For all bioreactor experiments, the pH was controlled at 6.7 using 15% (w/v) NH_4_OH, and the temperature was controlled at 37°C.

### Analyses

The optical density at 600 nm (OD) (UV-650 spectrophotometer, Beckman Instruments, San Jose, Calif.) was used to monitor cell growth, and this value was correlated to dry cell mass. Previously described liquid chromatography methods were used to quantify xylose and glucose [10] and other organic compounds [11].

The fraction of the microbial population that constituted each strain was determined by plating serial dilutions of cultures onto both LB and LB-tetracycline plates.

## Results

### Aerobic Utilization of Xylose/Glucose Mixtures

*Escherichia coli *ZSC113 and *E. coli *ALS1008 are unable to consume glucose and xylose, respectively. The xylose-selective strain ZSC113 has mutations in the three genes involved in glucose uptake [12], rendering it unable to consume glucose: *ptsG *codes for the Enzyme IICB^Glc ^of the phosphotransferase system (PTS) for carbohydrate transport [13], *manZ *codes for the IID^Man ^domain of the mannose PTS permease [14], *glk *codes for glucokinase [12]. We constructed strain ALS1008 which has a knockout in the *xylA *gene encoding for xylose isomerase, rendering ALS1008 unable to consume xylose. In a medium composed of a mixture of these two sugars, ZSC113 would be expected to consume the xylose selectively while ALS1008 should exclusively consume the glucose. We first sought to verify these expectations in three aerobic batch experiments.

**Figure 1:**
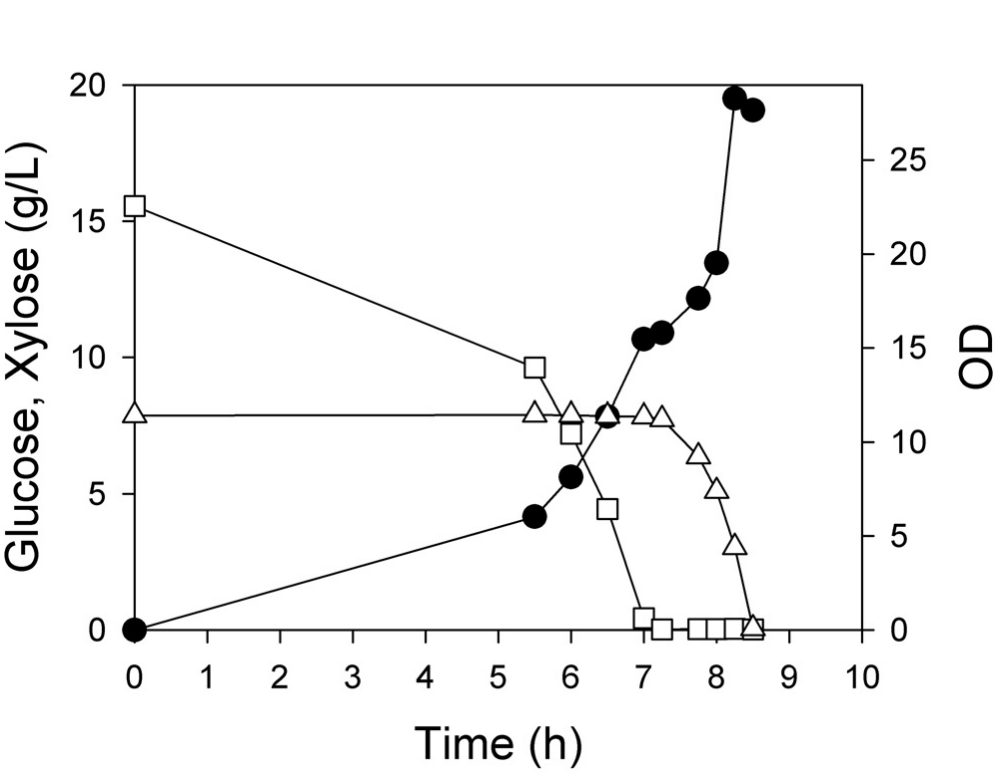
**Batch aerobic culture of *E. coli *MG1655**. Glucose (hollow square), xylose (hollow triangle), and OD (solid circle) were measured over the course of fermentations.

In a first (control) experiment, a defined medium containing both 8 g/L xylose and 15 g/L glucose was inoculated with a single wild-type strain, MG1655, and grown aerobically (Figure 1). The glucose/xylose mixture was chosen to reflect the concentrations of glucose and xylose that are found in typical lignocellulosic hydrolysates. As expected, we observed diauxic growth as reported by many other studies when a single strain is inoculated into a medium containing two or more carbon sources. The important observations were that glucose and xylose were consumed sequentially, and that the complete consumption of this mixture required about 8.5 hours.

**Figure 2:**
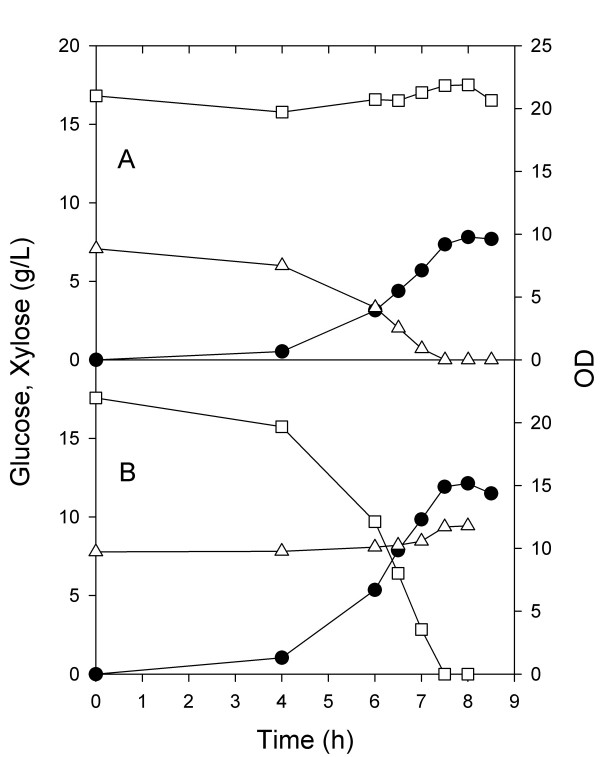
**Batch aerobic culture of individual substrate-selective *E. coli *strains**. Glucose (hollow square), xylose (hollow triangle), and OD (solid circle) were measured over the course of fermentations inoculated with A) ZSC113 only or B) ALS1008 only.

In a second set of aerobic experiments, the same defined medium containing two carbon sources was inoculated with one or the other of the two strains, ZSC113 or ALS1008. In the fermenter inoculated with only ZSC113 (Figure 2a), 8 g/L xylose was completely consumed in 7 h and the OD reached 10, In this case, the concentration of glucose remained unchanged. In the fermenter containing only ALS1008 (Figure 2b), we observed the complete consumption of 15 g/L glucose in 7.5 h with the OD reaching 15, while the concentration of xylose remained unchanged. As expected the two strains each consumed only one of the sugars, leaving the other carbohydrate unconsumed.

**Figure 3:**
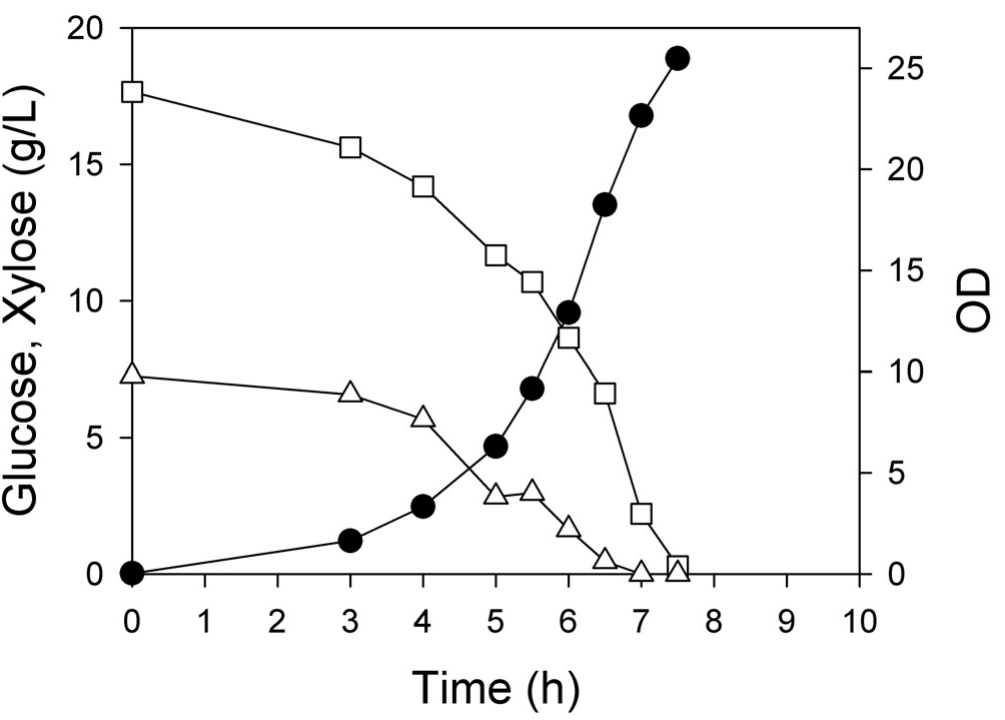
**Batch aerobic co-culture of two *E. coli *strains**. ZSC113 and ALS1008 were grown simultaneously on a mixture of glucose (hollow square) and xylose (hollow triangle). The OD (solid circle) was measured over the course of the fermentation.

In a third aerobic batch experiment, we inoculated both ZSC113 and ALS1008 into a single fermenter containing 8 g/L xylose and 15 g/L glucose. For this co-culture, glucose was consumed in 7.5 h, and xylose was simultaneously consumed in 7.0 h (Figure 3). Moreover, the final OD of this mixed culture was about 25, identical to the sum of the ODs achieved in the fermentations in which one or the other carbohydrate was used. Thus, each strain appears to grow and consume its substrate independently. The combined process (i.e. consuming both sugars simultaneously) occurred at the same rate as the two individual processes so that each consumption rate was unaffected by the presence of the other carbohydrate. Compared to the wild-type (single organism) process, this process required about 15% less time to consume the same carbohydrate mixture aerobically, and moreover each substrate was consumed independently. The single-organism process (Figure 1) was completely different than the dual-organism process (Figure 3) in which both carbon sources were consumed simultaneously. No products were observed in these batch fermentations.

### Aerobic Fed-Batch Utilization of Xylose/Glucose Mixtures

When a microorganism grows in a substrate-limited fashion (e.g. in a fed-batch process), the growth rate is controlled by the rate that the limiting substrate is supplied. Moreover, the concentration of that substrate remains at zero. In a bioprocess with two substrate-selective organisms which are both under carbon-limiting conditions, each organism should independently be controlled by and adapt to the quantity of the carbon source present that it can consume. We wished to test this hypothesis using a fed-batch process in which the two-carbohydrate feed increased exponentially at a nominal rate of 0.1 h^-1^, far below the maximum growth rate of either strain. Moreover, in addition to the flowrate exponentially increasing to maintain a fixed specific growth rate, the composition of the feed changed in discrete shifts in order to simulate a variable concentration that might be encountered in a real process. Specifically, for the first 20 h we maintained feed concentrations at 20 g xylose/L and 30 g glucose/L (20:30). At 20 h, this feed was replaced by feed concentrations of 30:30, at 30 h to 30:60, and then finally to 20:60 at 40 h. At 20 h, 30 h, 40 h and 50 h, we determined the fraction of the population which was the glucose-consuming strain ALS1008 (and thus by difference the fraction which was the xylose-consuming strain ZSC113).

**Figure 4:**
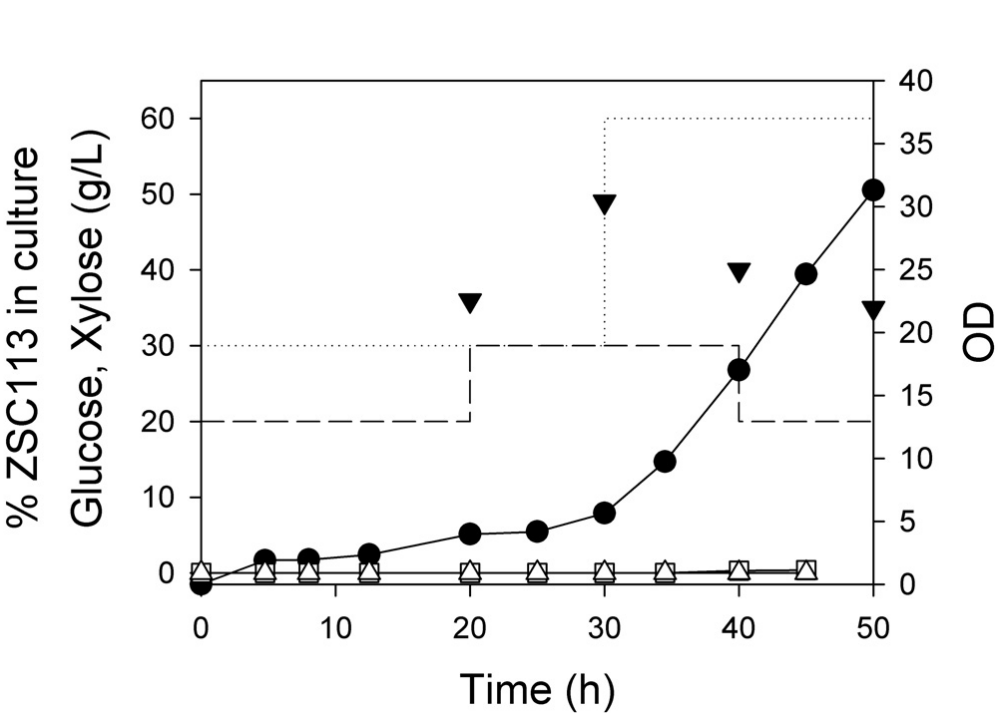
**Fed-batch aerobic co-culture of two *E. coli *strains**. ZSC113 and ALS1008 were grown simultaneously using a feed containing a varying mixture of glucose (dotted lines) and xylose (dashed lines). The OD (solid circle), the concentrations of glucose (hollow square) and xylose (hollow triangle), and the fraction of the total cell population which is ZSC113 (solid triangle pointing down) were measured over the course of the fermentation.

During the entire fed-batch process, the xylose and glucose concentrations in the fermenter remained at zero (Figure 4), demonstrating that each substrate individually limited the process. Moreover, the distribution of the microbial population responded in unison with the shift in substrate concentrations. At 20 h, after the process had acclimated to a 20:30 xylose:glucose composition (g/L), the population was 35% ZSC113 (i.e., the xylose-consuming strain). Ten hours after the feed composition shifted to 30:30, the population was 50% ZSC113. Similarly, ten hours after the feed composition shifted to 30:60, the population returned to 42% ZSC113, and then ten hours after the feed had become 20:60, the population decreased to 32% ZSC113. These results demonstrate that the process adjusts the distribution of strains to match the distribution of substrates.

### Anaerobic Product Formation with Xylose/Glucose Mixtures

Wild-type *E. coli *is a mixed acid fermenter, and generates acetate, lactate, formate, ethanol and succinate under anaerobic conditions, with the yield of each depending on the strain and carbon source [15]. ZSC113 and ALS1008 do not have any additional mutations which would cause the product distribution to be different from the wild-type parent MG1655. Three experiments were conducted under anaerobic conditions, analogous to those conducted under aerobic conditions previously.

**Figure 5:**
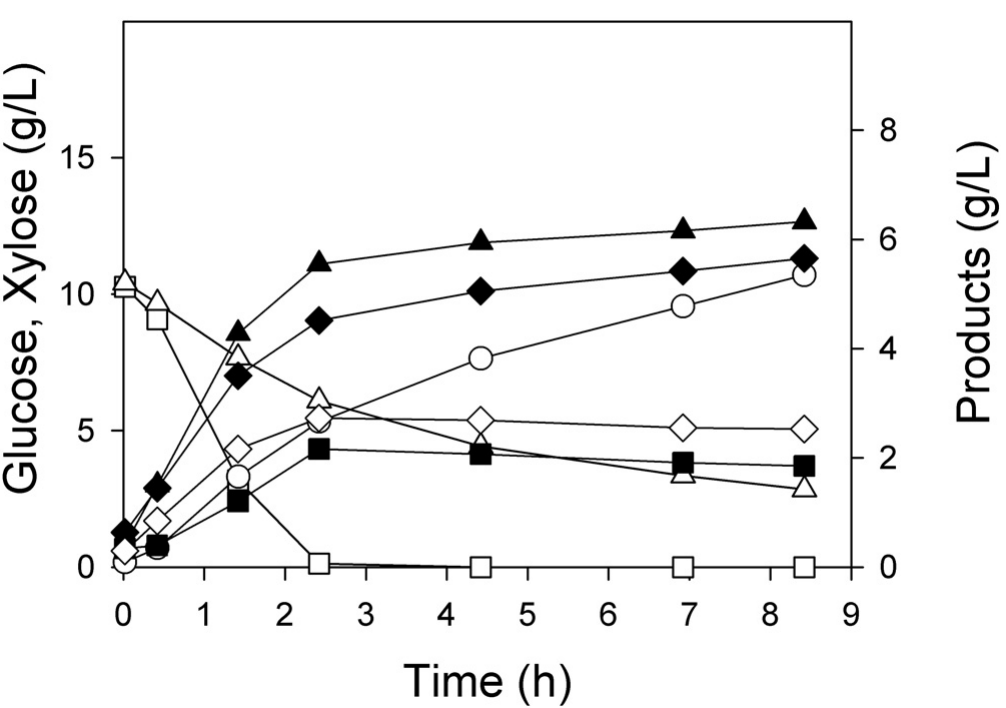
**Batch anaerobic fermentation of *E. coli *MG1655**. After aerobic growth, additional xylose (hollow triangle) and glucose (hollow square) were added and anaerobic conditions commenced (t = 0). The concentrations of formate (solid triangle), lactate (solid square), succinate (hollow circle), acetate (solid diamond) and ethanol (hollow diamond) were measured over the course of the anaerobic phase.

In a first experiment, wild-type MG1655 was grown under aerobic conditions as before to consume 15 g/L glucose and 8 g/L xylose. When both substrates were nearly consumed (after about 8.5 h as shown in Figure 1), we added enough glucose and xylose into the fermenter approximately to return both sugar concentrations to their initial levels. Anaerobic conditions were initiated under an atmosphere of 100% CO_2_, and the products were measured during the anaerobic growth phase (Figure 5). In this culture of a single strain (having an OD of 21), 10 g/L of glucose was consumed in about 2.5 h (4 g/Lh), equivalent to a specific glucose consumption rate of 630 mg/gh. Initially xylose was consumed at a rate of 310 mg/gh (2 g/Lh). However, after 4 h of anaerobic conditions, the xylose consumption rate decreased to less than 150 mg/gh (1 g/Lh), and continued to slow. Nearly 4 g xylose/L remained after 9 h of anaerobic conditions. It must be noted that the organism consumed glucose then xylose in the aerobic phase preceding these anaerobic conditions (as shown in Figure 1), and therefore at the time of the switch to anaerobic conditions, the xylose-consuming pathways were fully induced. One explanation for the substantial decrease in xylose consumption rate is the sensitivity of xylose-degradation to the presence of acetate as previously reported for yeast [16,17]. This explanation is supported by the observation that xylose consumption continued to slow even after glucose was depleted under anaerobic conditions.

**Figure 6:**
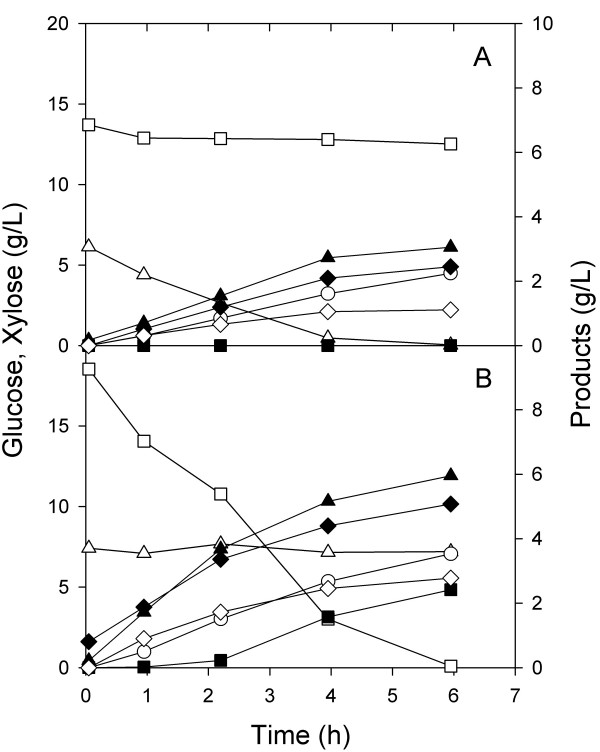
**Batch anaerobic fermentation of individual substrate-selective *E. coli *strains**. After aerobic growth, xylose (for ZSC113) or glucose (for ALS1008) was added and anaerobic conditions commenced (t = 0). The concentrations of glucose (hollow square), xylose (hollow triangle), formate (solid triangle), lactate (solid square) succinate (hollow circle), acetate (solid diamond) and ethanol (hollow diamond) were measured over the course of the anaerobic phase previously inoculated with A) ZSC113 only and B) ALS1008 only.

In a second experiment, the two substrate-selective strains ALS1008 and ZSC113 were grown individually on the mixed substrate medium, the one depleted substrate added back, and then anaerobic conditions commenced. For the case of the xylose-consuming strain ZSC113, xylose was consumed at a constant rate of 1.4 g/Lh during the anaerobic phase and glucose was not consumed (Figure 6a). This single organism was present only at an OD of 9.5, so that on a specific basis the xylose consumption rate was 500 mg/gh, greater than the highest rate observed in the xylose portion of the fermentation using the wild-type MG1655. For the experiment in which the glucose-consuming strain ALS1008 was inoculated into the mixed substrate medium, glucose was exclusively consumed during the anaerobic phase at a constant rate of 3 g/Lh, and xylose was not consumed (Figure 6b). In this case, the specific glucose consumption rate was about 770 mg/gh, greater than the rate we observed for the wild-type MG1655 during the anaerobic phase (i.e., Figure 5). These two separate fermentations demonstrate that the strains will each consume only one substrate under anaerobic conditions and that they will consume this substrate slightly faster on a specific basis than the wild-type strain would under identical conditions.

**Figure 7:**
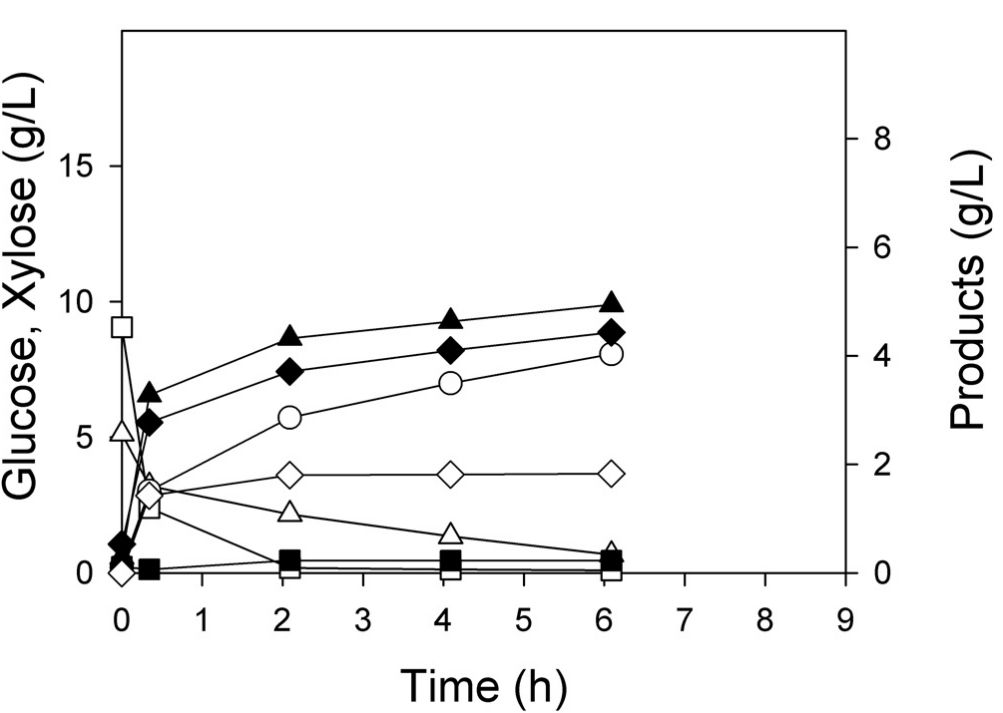
**Batch anaerobic co-fermentation of *E. coli *strains**. ZSC113 and ALS1008 were grown simultaneously on a mixture of glucose (□) and xylose (△). After aerobic growth, xylose and glucose were added and anaerobic conditions commenced (t = 0). The concentrations of formate (solid triangle), lactate (solid square), succinate (hollow circle), acetate (solid diamond) and ethanol (hollow diamond) were measured over the course of the anaerobic phase.

In a third experiment, we first simultaneously grew both strains in the mixed substrate medium under aerobic conditions. At the end of the 7.0 h aerobic growth phase, we added enough of both carbohydrates to return them to their initial concentrations, and anaerobic conditions were initiated. In this process, both xylose and glucose were quickly consumed (Figure 7). Although we did not measure the proportion of the two strains, from previous aerobic results using one substrate (i.e., Figure 2), we estimate that the OD of ZSC113 was about 13 and the OD of ALS1008 was about 9. Over the first two hours of the anaerobic phase, the xylose consumption rate was therefore about 475 mg/gh, while the glucose consumption rate was about 1300 mg/gh. The key point in these results is that the two-strain process is much faster than an otherwise identical single-strain process.

The products formed from the two-sugar fermentation were the same as those generated during either one of the single-sugar fermentations, although the distribution of products changed slightly. For example, the succinate yield from xylose using ZSC113 was 0.48 mol/mol, while the succinate yield from glucose using ALS1008 was 0.30 mol/mol. From the sugar mixture, the observed succinate yield by two organisms was 0.41 mol/mol sugar consumed, a value between the yields of the individual strains on the two substrates. For all three cases, formate was generated with the highest yield (1.24 mol/mol glucose, 1.58 mol/mol xylose, and 1.46 mol/mol sugar mixture), and lactate was generated the least.

One limitation of this study was that high concentrations of acid products are known to inhibit growth and substrate consumption rates. This phenomenon would tend to affect the mixed culture more than either single-sugar culture, since for the former case both sugars would quickly be converted into more mixed acid products. This substrate-selective approach may perform significantly better for strains specifically designed to accumulate a single product such as ethanol which does not cause acid inhibition.

## Discussion

The process described in this study offers a new approach for the simultaneous conversion of sugar mixtures into microbial products such as ethanol. The key characteristic of this approach is the use of multiple strains which are each selective in their consumption of a carbon source. Excluding substrate consumption in a strain by gene deletions represents an innovative shift from the long-studied approach of constructing a "do-it-all" organism for the conversion of multiple substrates into a desired product.

There are two significant advantages that the process has for the simultaneous conversion of sugar mixtures, as exemplified by xylose and glucose. Most importantly, as demonstrated by the fed-batch process (Figure 4), the system adapts to fluctuations in the feed stream, i.e. cultures actually grow in concert with the feed composition. Since they are each specialists, the strains can not adversely compete with each other for the consumption of the substrates. Using a fed-batch process prevents sugar accumulation, and permits each strain to convert its target sugar at high yield and productivity. Operational robustness is the hallmark of this process strategy, and it constitutes a major advance toward the utilization of lignocellulosic biomass. Second, although not part of this study, additional metabolic engineering strategies can focus on improving the individual production strains independently. For example, work can now be devoted to improving the glucose-selective strain for ethanol production with minimal concern for how these changes would impact the conversion of xylose. We do not need to compromise one objective for another.

Gene knockouts affecting only one carbohydrate consumption pathway appeared not to have deleteriously impacted the consumption of the other carbohydrate. Indeed, previous results have demonstrated improved xylose utilization in sugar mixtures by the *ptsG *knockout alone [18,19]. In this study, catabolite repression due to the presence of glucose was made irrelevant by the use of two strains, since one cannot utilize glucose at all.

The results demonstrate that a population of substrate-selective strains, in which each individual strain only consumes a single sugar, is better able to metabolize a sugar mixture than a single strain consuming multiple sugars. Other than the mutations involving substrate consumption, the strains used for this study did not contain additional mutations which would cause them to generate a product preferentially. The next step would be to use this approach with microbial strains specifically modified to accumulate a desired product such as ethanol. This approach could potentially be extended to construct additional strains capable of the exclusive consumption of other sugars (e.g., arabinose) or inhibitors such as acetic acid and furfurals that are frequently found in lignocellulosic hydrolysates.

## Competing interests

The author(s) declare that they have no competing interests.

## Authors' contributions

MAE, SAL and EA contributed to the design of the study. MAE and EA contributed to the writing of the manuscript. SAL conducted the fermentation experiments. All authors read and approved the final manuscript.

## References

[B1] Zaldivar J, Nielsen J, Olsson L (2001). Fuel ethanol production from lignocellulose: a challenge for metabolic engineering and process integration. Appl Microbiol Biotechnol.

[B2] Ho NWY, Chen Z, Brainard A (1998). Genetically engineered *Saccharomyces *yeast capable of effective cofermentation of glucose and xylose. Appl Environ Microbiol.

[B3] Sedlak M, Edenberg HJ, Ho NWY (2003). DNA microarray analysis of the expression of the genes encoding the major enzymes in ethanol production during glucose and xylose co-fermentation by metabolically engineered *Saccharomyces*. Enzyme Micro Technol.

[B4] Dien BS, Nichols NN, Bothast RJ (2002). Fermentation of sugar mixtures using *Escherichia coli *catabolite repression mutants engineered for production of L-lactic acid. J Industr Microbiol.

[B5] Barbosa MFS, Geck MJ, Fein JE, Potts D, Ingram LO (1992). Efficient fermentation of *Pinus *sp. acid hydrolysates by an ethanologenic strain of *Escherichia coli*. Appl Environ Microbiol.

[B6] Chandrakant P, Bisaria VS (2000). Simultaneous bioconversion of glucose and xylose to ethanol by *S. cerevisiae *in the presence of xylose isomerase. Appl Micro Biotechnol.

[B7] Dumsday GJ, Zhou B, Yaqin W, Stanley GA, Pamment NB (1999). Comparative stability of ethanol production by *Escherichia coli *KO11 in batch and chemostat culture. J Indust Micro Biotechnol.

[B8] Yu D, Ellis HM, Lee EC, Jenkins NA, Copeland NG, Court DL (2000). An efficient recombination system for chromosome engineering in *Escherichia coli*. Proc Natl Acad Sci USA.

[B9] Metcalf WW, Jiang W, Daniels LL, Kim SK, Haldimann A, Wanner BL (1996). Conditionally replicative and conjugative plasmids carrying *lacZ *alpha for cloning, mutagenesis, and allele replacement in bacteria. Plasmid.

[B10] Eiteman MA, Chastain MJ (1997). Optimization of the ion-exchange analysis of organic acids from fermentation. Anal Chem Acta.

[B11] Chesson A, Gordon AJ, Lomax JÅ (1993). Substituent groups linked by alkali labile bonds to arabinose and xylose residues of legume grass and cereal straw walls and their fate during digestion by rumen microorganisms. J Sci Food Agric.

[B12] Curtis SJ, Epstein W (1975). Phosphorylation of D-glucose in *Escherichia coli *mutants defective in glucosephosphotransferase, mannosephosphotransferase, and glucokinase. J Bacteriol.

[B13] Postma PW, Lengeler JW, Jacobson GR (1993). Phosphoenolpyruvate:carbohydrate phosphotransferase systems of bacteria. Microbiol Rev.

[B14] Huber F, Erni B (1996). Membrane topology of the mannose transporter of *Escherichia coli *K12. Eur J Biochem.

[B15] Clark DP (1989). The fermentation pathways of *Escherichia coli*. FEMS Microbiol Rev.

[B16] Helle S, Cameron D, Lam J, White B, Duff S (2003). Effect of inhibitory compounds found in biomass hydrolysates on growth and xylose fermentation by a genetically engineered strain of *S. cerevisiae*. Enzyme Microb Technol.

[B17] van Zyl C, Prior BA, du Preez JC (1991). Acetic acid inhibition of D-xylose fermentation by *Pichia stipitis*. Enzyme Microb Technol.

[B18] Kimata K, Takahashi H, Inada T, Postma P, Aiba H (1997). cAMP receptor protei-cAMP plays a crucial role in glucseo-lactose dizuxie by activating the major glucosa transporter gene in *Escherichia coli*. Proc Natl Acad Sci USA.

[B19] Nichols NN, Dien BS, Bothast RJ (2001). Use of catabolite repression mutants for fermentation of sugar mixtures to ethanol. Appl Microbiol Biotechnol.

